# Evaluating the Efficacy of Automated Smoking Treatment for People With HIV: Protocol for a Randomized Controlled Trial

**DOI:** 10.2196/33183

**Published:** 2021-11-17

**Authors:** Damon J Vidrine, Thanh C Bui, Michael S Businelle, Ya-Chen Tina Shih, Steven K Sutton, Lokesh Shahani, Diana Stewart Hoover, Kristina Bowles, Jennifer I Vidrine

**Affiliations:** 1 Department of Health Outcomes and Behavior Moffitt Cancer Center Tampa, FL United States; 2 Stephenson Cancer Center TSET Health Promotion Research Center University of Oklahoma Health Sciences Center Oklahoma City, OK United States; 3 Department of Health Services Research The University of Texas MD Anderson Cancer Center Houston, TX United States; 4 Department of Biostatistics and Bioinformatics Moffitt Cancer Center Tampa, FL United States; 5 Hoover Editing Asheville, NC United States

**Keywords:** smoking cessation, health disparities, HIV/AIDS, mHealth, mobile phone

## Abstract

**Background:**

Smoking prevalence rates among people with HIV are nearly 3 times higher than those in the general population. Nevertheless, few smoking cessation trials targeting smokers with HIV have been reported in the literature. Efforts to develop and evaluate sustainable, low-cost, and evidence-based cessation interventions for people with HIV are needed. Given the widespread proliferation of mobile phones, the potential of using mobile health apps to improve the reach and efficacy of cessation interventions is promising, but evidence of efficacy is lacking, particularly among people with HIV.

**Objective:**

This study will consist of a 2-group randomized controlled trial to evaluate a fully automated smartphone intervention for people with HIV seeking cessation treatment.

**Methods:**

Participants (N=500) will be randomized to receive either standard treatment (ST; 250/500, 50%) or automated treatment (AT; 250/500, 50%). ST participants will be connected to the Florida Quitline and will receive nicotine replacement therapy in the form of transdermal patches and lozenges. This approach, referred to as Ask Advise Connect, was developed by our team and has been implemented in numerous health systems. ST will be compared with AT, a fully automated behavioral treatment approach. AT participants will receive nicotine replacement therapy and an interactive smartphone-based intervention that comprises individually tailored audiovisual and text content. The major goal is to determine whether AT performs better in terms of facilitating long-term smoking abstinence than the more resource-intensive ST approach. Our primary aim is to evaluate the efficacy of AT in facilitating smoking cessation among people with HIV. As a secondary aim, we will explore potential mediators and moderators and conduct economic evaluations to assess the cost and cost-effectiveness of AT compared with ST.

**Results:**

The intervention content has been developed and finalized. Recruitment and enrollment will begin in the fall of 2021.

**Conclusions:**

There is a critical need for efficacious, cost-effective, and sustainable cessation treatments for people with HIV who smoke. The AT intervention was designed to help fill this need. If efficacy is established, the AT approach will be readily adoptable by HIV clinics and community-based organizations, and it will offer an efficient way to allocate limited public health resources to tobacco control interventions.

**Trial Registration:**

ClinicalTrials.gov NCT05014282; https://clinicaltrials.gov/ct2/show/NCT05014282

**International Registered Report Identifier (IRRID):**

PRR1-10.2196/33183

## Introduction

### HIV and Cigarette Smoking

In the United States, the prevalence of cigarette smoking in the general population has dropped to 13.7% [1]; however, smoking rates among people with HIV remain quite high, with evidence suggesting that 34% to 42% are current smokers [2,3]. Thus, smoking among people with HIV is a leading cause of morbidity and mortality [4-7]. People with HIV who smoke are more likely to die of lung cancer than AIDS-related diseases, even after accounting for antiretroviral therapy adherence [8]. They are also at greater risk of experiencing various oral, renal, and cardiovascular diseases, decreased bone mineral density and fracture, pulmonary complications, tuberculosis, opportunistic and nonopportunistic infections, and poor quality of life [7,9-12]. Moreover, smoking negatively affects the response to antiretroviral therapies, resulting in poor viral and immunologic response [13]. Among people with HIV, the mortality rate for smokers is twice that of nonsmokers [5,14], and the estimated population-attributable risk of all-cause mortality associated with smoking ranges from 24% to 62% [4,6]. Notably, smoking-related morbidity and mortality appear to decline over time in former versus current smokers with HIV [5,15]. Effective smoking cessation treatment is critical for lowering HIV- and smoking-related morbidity, improving antiretroviral therapy response, and reducing overall mortality.

### Smoking Cessation Treatment for HIV-Positive Smokers

Despite this need, few studies have evaluated the efficacy of smoking cessation interventions targeting people with HIV [15]. Studies have shown that people with HIV who smoke are interested in quitting and receptive to cessation interventions [2,16,17], and smoking treatment programs have been successfully implemented in HIV clinics [18]. Although a recent systematic review found that smoking cessation interventions for people with HIV were effective at short- and intermediate-term follow-up (ie, 3-month), limited evidence supports long-term (ie, 6- and 12-month) effectiveness [19]. The results from our prior work are in line with these conclusions. Most eligible people with HIV who smoked (roughly two-thirds) enrolled in treatment, and those randomized to receive our interventions (vs controls) had higher abstinence rates through the 3-month follow-up. However, long-term relapse rates were high, with treatment effects diminished by the 6-month follow-up [20,21]. Efforts are needed to elucidate the resources needed to engage people with HIV who smoke during treatment and facilitate long-term abstinence.

### Quitline-Delivered Smoking Cessation Treatment in Vulnerable Populations

Our team developed an approach to link smokers in health care settings with evidence-based treatment delivered via state quitlines. This approach—Ask Advise Connect (AAC)—links smokers with treatment via an automated connection system. Results from efficacy trials revealed that AAC was associated with a 13- [22] to 30-fold [23] increase in treatment enrollment when compared with Ask Advise Refer, where smokers were offered a quitline referral and encouraged to call on their own. We have successfully implemented AAC in various settings (eg, safety-net hospitals and HIV clinics) throughout Texas and Oklahoma [24,25].

While the results are encouraging, several factors suggest that interventions such as AAC that depend on connecting smokers with quitlines may not be sufficient. First, in recent years, state quitlines have experienced significant budget cuts, and some states have temporarily eliminated quitlines altogether [26-28]. Moreover, while quitlines provide a cost-effective and evidence-based treatment option and have the potential to reach countless smokers [29], human-delivered phone counseling has limited appeal. In fact, national data suggest that quitlines reach only 1% to 2% of smokers [30]. Finally, results from a recent AAC implementation study indicated that self-reported abstinence was 18.7% among HIV clinic patients and 16.5% among non–HIV clinic patients; however, biochemically confirmed abstinence was considerably lower (4.2% and 4.5%, respectively) [24,25]. There is a critical need to improve the reach, efficacy, sustainability, and impact of smoking cessation treatment for people with HIV [31].

### Mobile Technology and Smoking Cessation Treatment

Over the last 20 years, cell phone ownership has steadily increased. The Pew Research Center found that as of February 2021, 97% of adults living in the United States reported owning a cell phone [32]. Previous studies have used cell phones to administer text message–based smoking cessation interventions, and findings suggest excellent reach and efficacy in the general population [33-35] and among people with HIV who smoke [36]. Moreover, text message–based interventions are cost-effective [37,38] and affordable options for global tobacco control [39].

Compared with cell phones, smartphones have greater capability, as they can be used to access the internet, run apps, view and send graphic messages, and stream audio and video content. According to Pew, in 2021, 85% of US adults reported owning a smartphone [32]. Smartphone ownership is high among adults between the ages of 18 and 64 years (83%) and within underserved populations, such as racial or ethnic minorities (83%), individuals with less than a high school education (75%), and those with an annual household income of less than US $30,000 (76%). Notably, the proportion of individuals who depend on smartphones for all internet access is higher among racial and ethnic minority groups (vs White individuals), those with lower income and education, and individuals living in rural (vs urban and suburban) communities.

Current national trends indicate that US smartphone ownership is nearing ubiquity. Smartphone-delivered interventions are an ideal method for reaching underserved populations (eg, minority, low income, low education, and rural). Although numerous smartphone-delivered smoking cessation apps exist, few were constructed using evidence-based practices, and there is little outcome data to support the efficacy of these treatments [40,41]. Theoretically grounded smartphone-based smoking cessation interventions have broad scalability and dissemination potential and are likely to be cost-effective. Efforts are needed to evaluate these treatments in underserved populations, particularly among people with HIV who smoke.

### Objectives

This paper describes the protocol for a randomized controlled trial (RCT) that will assess the efficacy of a fully automated smartphone intervention for people with HIV who smoke. Participants will be randomized to receive either (1) standard treatment (ST) or (2) automated treatment (AT). ST participants will be connected to the Florida Quitline (AAC, which was developed and evaluated by our team) and will receive nicotine replacement therapy (NRT) in the form of transdermal patches and nicotine lozenges. ST will be compared with AT, a fully automated behavioral treatment approach. AT participants will receive NRT plus an interactive smartphone-based intervention that comprises individually tailored audiovisual and text content.

Our primary aim is to evaluate the efficacy of AT in facilitating smoking cessation among people with HIV. We expect that at 12 months postenrollment, smoking abstinence rates will be higher in the AT group than in the ST group. Regarding secondary aims, we will first explore potential mediators and moderators. We will compare the magnitude of the mediated effects via common mechanisms (ie, motivation, agency, and stress or negative affect) on smoking abstinence between the AT and ST treatment groups. We will also examine the role of HIV-specific moderators (ie, stigma, resilience, disease progression, and HIV symptom burden). Second, we will evaluate the cost and cost-effectiveness of AT versus ST. Through these aims, we will determine whether AT performs better in terms of facilitating long-term smoking abstinence (ie, 12 months postenrollment) than the more resource-intensive ST approach. If efficacy is established, the AT approach will be readily adoptable by various HIV clinics and community-based organizations and offer an efficient way to allocate limited public health resources to tobacco control interventions.

## Methods

### Design Overview

This study will use a 2-group RCT to compare ST with AT. We will enroll a total of 500 participants (250/500, 50% per group). An additional 20 participants (10 per group) will be enrolled in a 12-week pilot study before the implementation of the full trial. All participants will be recruited on the web from Florida. Participants will complete assessments on the web via the Research Electronic Data Capture (REDCap) platform (Vanderbilt University) hosted at H. Lee Moffitt Cancer Center and Research Institute [42,43] or over the phone at baseline and at 3, 6, and 12 months postenrollment. These assessments will take approximately 20 minutes to complete. Weekly 4-item smartphone assessments will be collected from all participants for 26 weeks.

### Eligibility Criteria

The eligibility is determined based on inclusion and exclusion criteria (Textbox 1).

**Inclusion and exclusion criteria.**

**Inclusion criteria**

- At least 18 years of age
- Smoked at least 100 cigarettes in their lifetime
- Currently smoking at least five cigarettes a day
- Willing to make a quit attempt within 1 week of enrollment
- HIV-positive
- English or Spanish speaking
- Possess a smartphone with a data plan and operating system compatible with the project app
- Have a valid email address

**Exclusion criteria**

- Medical conditions that preclude the use of nicotine replacement therapy
- Current use of smoking cessation medications
- Enrollment in another cessation study
- Household members enrolled in the study
- Inadequate health literacy

### Recruitment and Screening

Participants will be recruited from Florida using web-based advertisements. As of 2018, 116,689 Floridians were living with HIV [44]. On the basis of national data suggesting that 32% to 42% of US adults with HIV currently smoke [2,3], we expect that about 39,675 will be current smokers. Thus, our recruitment goal of 500 participants should be achievable within the proposed 42-month recruitment period.

Web-based advertisements will describe the study and direct potential participants to a landing page, which will provide more information about the study. Interested participants will complete a short web-based prescreening questionnaire, and those who pass the prescreener will be automatically redirected to the study’s eligibility questionnaire. Those who do not complete the self-administered web-based screening form will be contacted by the study staff and offered screening over the phone. Individuals who are eligible and interested in participating in the study will provide informed consent electronically or verbally over the phone and receive a copy of the informed consent document via email or mail. They will then provide HIV status documentation via a REDCap link. Once the documentation is verified, participants will be asked to complete a baseline assessment. The full list of measures to be completed for each assessment is shown in Table 1.

**Table 1:** Study measures and assessment schedule.

Measure	Assessment schedule				
	Baseline	Weekly	3-month	6-month	12-month
Demographics and smoking history [45,46]	✓				
Drug use history [47]	✓				
Alcohol history and follow-up [48]	✓		✓	✓	✓
Smoking status [49]		✓^a^	✓	✓	✓
Heaviness of smoking index [50]	✓				
Financial Strain Questionnaire [51]	✓		✓	✓	✓
Contemplation ladder [52]	✓	✓^a^	✓	✓	✓
Reasons for quitting (intrinsic and extrinsic motivation) [53]	✓				
Sense of control [54]	✓		✓	✓	✓
Self-efficacy Scale [55]	✓	✓^a^	✓	✓	✓
Perceived Stress Scale-4 [56]	✓	✓^a^	✓	✓	✓
Positive and Negative Affect Scale [57,58]	✓		✓	✓	✓
Patient Health Questionnaire-8 [59]	✓		✓	✓	✓
Client Satisfaction Questionnaire [60]			✓	✓	
Health utilities (EQ-5D-5L^b^) [61]	✓		✓	✓	✓
Antiretroviral therapy adherence [62]	✓		✓	✓	✓
HIV symptoms burden [63]	✓		✓	✓	✓
Brief Resilience Scale [64]	✓		✓	✓	✓
HIV stigma scale [65]	✓		✓	✓	✓
Clinical measures	✓				✓
USDA^c^ household food insecurity survey [66]	✓		✓	✓	✓
CDC^d^ healthy days core module [67,68]	✓		✓	✓	✓
Subjective social status [69]	✓		✓	✓	✓
Three-item Loneliness Scale [70]	✓		✓	✓	✓
Brief Everyday Discrimination Scale [71]	✓		✓	✓	✓
COVID-19 risk perception, testing, and vaccination history	✓		✓	✓	✓
PROMIS^e^ global health items [72]	✓		✓	✓	✓
HPV^f^ and hepatitis B vaccination	✓				✓
Cancer screenings (cervical, colorectal, lung)	✓				✓
Brief weekly survey		✓			
Cotinine survey			✓	✓	✓

^a^Denotes brief version.

^b^EQ-5D-5L: EuroQol Five Dimension Five Level Scale.

^c^USDA: United States Department of Agriculture.

^d^CDC: Centers for Disease Control and Prevention.

^e^PROMIS: Patient-Reported Outcomes Measurement Information System.

^f^HPV: human papillomavirus.

### Randomization

Following completion of the baseline assessment, the participants will be randomized to the treatment group (ST or AT) using stratified randomization. Sex assigned at birth, HIV disease stage, and nicotine dependence will be balanced.

### Participant Tracking, Compensation, and Retention Procedures

We will use various approaches to maximize follow-up rates. The baseline and the 3-, 6-, and 12-month assessments will be conducted on the web or over the phone at participants’ convenience. Participants will be compensated for up to US $160 for completing these assessments (4 assessments × US $40 = US $160). Compensation for study-related smartphone use (ie, data, texting, and minutes) will be provided monthly for 26 weeks, based on the number of weekly assessments completed (26 weekly assessments × US $10 = US $260). We will also compensate participants for returning cotinine tests at 3, 6, and 12 months (3 tests × US $30 = US $90). Other procedures to reduce attrition will include (1) reminder phone calls/messages delivered via the app or by study staff before the follow-up assessments; (2) obtaining the names, addresses, and phone numbers of 3 collaterals (ie, relatives and friends) who can provide information on participants’ whereabouts; and (3) using the Whitepages website to search for updated participant contact information on the web.

### Conceptual Framework

**Figure 1:**
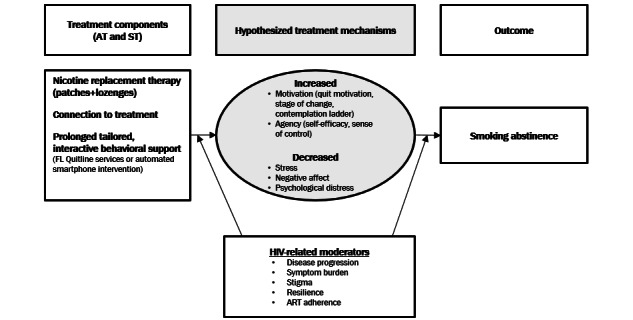
Hypothesized mechanisms underlying standard treatment and automated-treatment. ART: antiretroviral therapy; AT: automated treatment; FL: Florida; ST: standard treatment.

Research and theory have identified motivation and agency as critical mechanisms underlying the decision to enroll in smoking cessation treatment, and motivation, agency, and stress or negative affect are established mechanisms underlying successful cessation [73-80]. As such, both ST and AT are designed to target these mechanisms. Although we hypothesize common mechanisms between ST and AT, we do not expect equivalent magnitudes of the effect. Thus, a secondary aim is to compare the magnitude of the mediated effects between AT and ST. For example, participants in ST (vs AT) may report higher quit motivation, whereas individuals in AT (vs ST) may report higher self-efficacy. See Figure 1 for the conceptual framework.

ST is intended to mirror a clinic-based AAC model, which involves delivery of brief advice to quit video followed by an invitation to enroll in treatment that mirrors standard proactive quitline-delivered phone counseling. AT is designed to function as a fully automated program capable of delivering cessation treatment via automated, smartphone-based treatment content that comprises an initial brief advice to quit video, followed by interactive messaging, images, and audiovisual clips. We have elected to extend AT over a 6-month period (vs a more conventional 3 months) to counter the high relapse rates among people with HIV who smoke observed in our previous studies [20,21]. All participants will receive NRT.

#### Motivation

Motivation refers to an individual’s willingness and desire to make a specific change in their behavior [81]. Substantial evidence underscores the critical role of motivation in the process of decision-making regarding change and the likelihood of achieving and successfully maintaining change [73,74], as *stage of change* predicts both quit attempts and cessation success [73-75]. Notably, the motivation for change can fluctuate rapidly [82,83]. In fact, 41% of US smokers report that their motivation to quit smoking changes daily [84], and half or more quit attempts are unplanned [85,86], consistent with a model of smoking motivation that posits that motivation is characterized by frequent fluctuations [86]. After the initial change has been achieved, motivation for maintaining abstinence may weaken, and ambivalence may increase as the individual is exposed to temptations and stressors [76]. Because empirical data indicate that motivation influences the initiation of a quit attempt, success in achieving cessation, and the maintenance of abstinence, efforts are needed to bolster individuals’ motivation during the process of quitting [53,83,87]. A crucial focus of both interventions is to provide an appropriate therapeutic response to fluctuations in motivation throughout the change process.

#### Agency (Sense of Control and Self-efficacy)

Human agency reflects the ability to intentionally affect one’s behavior or life situation; a sense of agency is determined both by personal resources and situational influences [88,89]. Agency includes constructs such as a sense of control and self-efficacy [89]. Sense of control refers to the learned expectation that outcomes depend on personal choices and actions rather than on chance, other people, or forces outside of one’s control [54,90]. Self-efficacy is a form of agency that is dependent on context and behavior [90,91]. In the context of smoking, agency is reflected in greater self-efficacy when faced with situations that challenge one’s ability to initiate or maintain abstinence. Self-efficacy is a strong predictor of cessation treatment outcomes [78,79,87,92]. Both AT and ST will target agency as a key mechanism.

#### Stress or Negative Affect or Psychological Distress

Stress/negative affect, measured in many ways, is associated with change. For example, Shiffman et al [80] found a strong dose-response relationship between smoking-related acute events and the severity of stress or negative affect. In addition, the magnitude and trajectory of stress or negative affect over time are powerful predictors of cessation [93,94], as are individual differences in affective vulnerability [95,96]. Results indicate that most smokers experience elevated levels of postcessation negative affect that continue for relatively long periods, regardless of NRT use [97]. Similarly, studies have shown that mood disorders, negative affect, and stress are associated with poorer treatment outcomes, including tobacco and alcohol relapse [92,98-101]. Given the multiple stressors (eg, medical, psychosocial, economical, and stigma) confronting people with HIV who smoke, we expect that addressing stress or negative affect in the context of smoking treatment will be crucial for this population [15,102].

#### Reciprocal Relations Among Mechanisms

Theory and data suggest reciprocal relationship among the key hypothesized mechanisms—motivation, self-efficacy, and stress or negative affect—targeted by the treatments (ST and AT) [76,92,103-105]. ST and AT are hypothesized to reduce stress or negative affect via the following mechanisms: (1) their impact on decreasing ambivalence and increasing motivation; (2) the application of coping skills training and problem solving to increase self-efficacy; and (3) the use of a holistic approach, where other life concerns and stressors will be addressed. Similarly, reductions in stress or negative affect are posited to increase motivation and agency.

#### HIV-Related Moderators

Our prior work and the extant literature suggest that several HIV-related variables may moderate the relationship between interventions and cessation outcomes. For example, we have observed higher quit rates and higher intention to quit among people with HIV with more advanced disease [106]. Conversely, we have observed lower cessation rates among people with HIV who report greater symptom burden and those with low adherence to antiretroviral therapies [107-109]. Finally, stigma is a risk factor for smoking among people with HIV, whereas resilience is associated with improved HIV-related outcomes [110,111].

### Treatment Groups

#### ST Group

Following the baseline assessment and brief advice to quit video, participants randomized to ST will be given a 10-week supply of nicotine patches and lozenges. Nicotine patches provide a low, constant level of nicotine, which attenuates nicotine withdrawal symptoms after quitting (including physical symptoms and negative affect), while lozenges provide a fast-acting dose of nicotine that can be used as needed to combat cravings. When combined with behavioral treatment, NRT doubles the odds of successfully quitting [112]. ST participants will also be connected to Florida Quitline services and complete weekly 4-item smartphone assessments for 26 weeks. Weekly assessments will measure smoking status, motivation, self-efficacy, and perceived stress.

#### AT Group

Following the baseline assessment and brief advice to quit video, participants in AT will be given a 10-week supply of nicotine patches and lozenges (equivalent to ST). After randomization, they will be sent an email with a link to the smartphone app and instructions on how to use the app. AT will include (1) 12 proactive treatment videos (delivered weekly) that will be tailored to smoking status, motivation, agency, and negative affect or stress; (2) 26 weeks of on-demand access to treatment content; and (3) 26 weeks of text content.

As with participants in the ST group, AT participants will complete weekly 4-item smoking status, self-efficacy, motivation, and stress assessments for 26 weeks. An algorithm will use responses to these assessments to deliver brief, 2- to 6-minute, videos from an existing library of tailored content. Weekly topics are listed in Table 2. The AT app will deliver the most appropriate video each week, specifically tailored for each participant. During the first 12 weeks, videos will be proactively launched each week following the completion of the assessment. Throughout the entire 26-week treatment period, participants will also have the option to self-initiate treatment sessions. In addition, referrals to extra treatment resources will be available through the app. Resources will include phone numbers to substance abuse treatment centers, psychiatric services, HIV case management, and other services (eg, housing needs).

**Table 2:** Automated treatment session topic by study week.

Study week	Topic
Week 1	Preparing to quit
Week 2	Quit day
Week 3	What to do if you have a cigarette
Week 4	Nicotine patches and lozenges
Week 5	Smoking, stress, and mood
Week 6	Health benefits of quitting
Week 7	Smoking and your weight
Week 8	Staying on track
Week 9	Financial benefits of being a nonsmoker
Week 10	Taking care of yourself
Week 11	Social benefits of being a nonsmoker
Week 12	Life without cigarettes

The primary goal of the AT app is to function autonomously. It is designed to require minimal human involvement while being appropriate for implementation in various environments. This approach is expected to perform better than, yet share common mechanisms of action (eg, motivation, agency, and stress or negative affect) with, the behavioral standard of care (ie, quitline counseling). Underlying AT is a platform consisting of both a staff-facing dashboard and a mobile app. Videos were created with Adobe After Effects, an animation software package that is widely used in media campaigns.

On the basis of our prior work, as well as the growing literature supporting the efficacy of text messaging interventions for promoting smoking cessation, participants in the AT group will also receive smartphone-delivered text content [39,94]. This content will be delivered in the form of notifications generated by the app. Daily notifications will begin shortly before the scheduled quit date and continue through month 3. During months 4 to 6, the notification frequency will drop to once per week. The content of these notifications will be designed to target the hypothesized mechanisms and promote NRT adherence. Furthermore, the notifications will encourage participants to access additional on-demand content within the app. Participants will be referred to by first name within the notifications as a way of personalizing the treatment.

### Measures and Assessment Strategy

Several considerations guided our assessment procedures. First, we attempted to include measures with established reliability and validity. If measures with established psychometric properties were not available, measures were required to at least have face validity. Second, assessments had to either (1) represent our hypothesized mechanisms, (2) empirically predict smoking behavior, or (3) describe the sample. Measures were informed by models of health behavior, theories of nicotine dependence, and existing data. To ensure that the study is available to a representative sample of the target population, we will recruit both English- and Spanish-speaking participants. Most of the measures have been translated and validated in Spanish. A certified translator will be used for measures that have not yet been translated.

The REDCap system will be used to administer the surveys. REDCap is a secure web-based app designed to support secure data capture for cleaning, storage, and analysis. Moffitt Cancer Center is a member of the REDCap Consortium.

#### Primary Outcomes

The main outcome is self-reported 7-day abstinence smoking status at the 12-month follow-up. Other definitions of abstinence, such as biochemically verified abstinence using salivary cotinine and self-reported 24-hour, 30-day, and continuous abstinence, will be examined as secondary outcomes. Participants who report 7-day abstinence will be mailed a cotinine kit with instructions for providing the cotinine sample. Study staff will be available by phone and email if participants have questions about the collection process. Participants will be asked to return cotinine samples using a prepaid envelope.

All participants (ST and AT) will complete brief smartphone-delivered assessments each week during the 26-week treatment period. These assessments will be delivered to AT participants through the app, while ST participants will receive their assessments through a REDCap link. Regardless of the treatment group, assessments will be identical and include current smoking status, motivation, agency, and stress or negative affect. Responses to the assessments will be used to select the appropriate treatment content for AT participants, as described above.

#### Assessment of Treatment Engagement–Related Variables in AT

An important focus of our evaluation of AT is to track and examine how frequently participants interact with the app and the duration of these interactions. Thus, each interaction will be date- and timestamped, and we will note how often specific features of the app are used. These data will allow us to examine the frequency and duration of participants’ use of the various components of the app and the specific conditions under which participants engage with the app components. This information will be used to help guide future refinements of the app.

### Data Analysis Plan

Descriptive analyses will be conducted to determine which variables, if any, should be transformed before inferential analyses. In addition, descriptive statistics for demographic, smoking-related, and health-related variables will be used to characterize each sample. The samples will be compared to identify differences despite randomization, which may necessitate inclusion in models testing hypotheses. Descriptive statistics will also be used to summarize the participants’ interactions with the app.

### Primary Aim

The primary aim of this study is to evaluate the efficacy of AT in facilitating smoking cessation among people with HIV. We hypothesize that at 12 months postenrollment, smoking abstinence rates will be higher in the AT group than in the ST group. The primary outcome is self-reported 7-day point prevalence abstinence at 12 months. As is traditional in smoking cessation research, participants who do not complete a follow-up will be coded as smoking. Log-binomial regression will be used, with the intervention group (AT vs ST) as the primary predictor. Variables found to differ by group in preliminary analyses (*P*<.10) will be included as covariates. Differences in treatment effects as a function of sex assigned at birth will also be examined.

Additional analyses will evaluate the effects of the intervention on other definitions of smoking abstinence: biochemically verified abstinence using salivary cotinine and self-reported 24-hour, 30-day, and continuous abstinence. Outcomes from the 3- and 6-month follow-ups will also be considered. Repeated-measures analysis using generalized linear mixed models with a log-link function and the appropriate random-effect covariance structure will be used to analyze outcomes across all assessments with intervention, time, and their interaction as the primary model variables. Model fitting and diagnostics will follow the general approach described by McCullagh and Nelder [113] and McCulloch et al [114], when applicable. Adjustments for multiple comparisons will be made using the methods recommended by Westfall and Young [115].

### Secondary Aim 1

The first secondary aim is to explore the role of potential mediators and moderators. Specifically, we will compare the magnitude of the mediated effects via common mechanisms (ie, motivation, agency, and stress or negative affect) on smoking abstinence between the AT and ST groups. We will also investigate the role of several established HIV-specific moderators (ie, stigma, resilience, disease progression, and HIV symptom burden). Furthermore, exploratory analyses will examine the associations of various AT treatment components with outcomes.

While we hypothesize common mechanisms of AT and ST, it is important to identify the relative strengths and weaknesses of the mechanisms on which each approach relies to facilitate cessation. Participants in ST (vs AT) may report higher levels of quit motivation, whereas those in AT (vs ST) may report higher self-efficacy. Such hypotheses can be tested via mediation analyses with the intervention group (AT vs ST) as the independent variable and smoking abstinence and the hypothesized mechanisms (ie, motivation, agency, and stress or negative affect) as potential mediators. Mediation will be evaluated using approaches developed by MacKinnon [116] and Preacher and Hayes [117-119]. We will also explore the role of HIV-specific moderators (ie, stigma, resilience, disease progression, and HIV-symptom burden) of these indirect effects on the outcome of smoking abstinence.

### Secondary Aim 2

Our second secondary aim is to conduct economic evaluations from a societal perspective as well as a health system perspective to evaluate the cost and cost-effectiveness of AT versus ST. Although the societal perspective is recommended by the Second Panel on Cost-Effectiveness in Health and Medicine [120], we expect that a health system perspective will be of greater interest to decision makers in the public health sector who are responsible for making implementation decisions about smoking cessation programs. Conventional cost-effectiveness analysis will be used to summarize findings in terms of the incremental cost-effectiveness ratio (ICER) [120-122]. The ICER, calculated as the difference in mean costs between new (ie, AT) and ST divided by the difference in mean effectiveness between the two, estimates the additional resources needed to achieve an increase in one unit of effectiveness. We will use 2 commonly used effectiveness measures—number of quitters and years of life saved (YOLS) [123], to compare the ICER with other published cost-effectiveness analyses. The number of quitters in each arm will be retrieved from the 12-month self-reported abstinence assessment. We will extrapolate from abstinence to YOLS using a published algorithm that models YOLS per quitter [124]. The algorithm will be revised using current estimates of age-specific smoking-attributable deaths [125]. We will also include quality-adjusted life-years, which will be calculated based on a measure of health utilities, the EQ-5D-5L [61].

We will also use the net benefit approach [126,127], which transforms the ICER into a net benefit, defined as NB(λ) = λ × ΔE – ΔC, where λ represents the societal willingness to pay, ΔC represents the incremental costs, and ΔE represents the incremental effectiveness. A benefit of the net benefit approach is that it can be incorporated into a regression framework to allow for covariate adjustments and the examination of interaction effects [128].

Moreover, we will assess the short- and long-term economic impact of the interventions. The short-term analysis will use the number of quitters and quality-adjusted life-years as the effectiveness measure and assess cost-effectiveness based on information collected at the 3-, 6-, and 12-month follow-ups. The long-term analysis will extrapolate the intervention effect to lifetime and use YOLS. A 3% discount rate will be applied to costs and outcomes accrued in the second year and forward. We will first perform a deterministic analysis, where point estimates of ICERs or cost differences will be calculated. To obtain the 95% CIs, we will apply nonparametric bootstrapping methods to the person-level data [129]. We will conduct one-way sensitivity analyses to examine the impact of alternative measures of cost and outcomes. We will then apply the Bayesian approach to construct the cost-effectiveness acceptability curve and conduct a probabilistic sensitivity analysis [130,131]. We will conduct Bayesian analysis using WinBUGS (University of Cambridge) or STATA (StataCorp LLC), with costs modeled as a gamma or log-normal distribution and abstinence as a binomial distribution. Finally, we will apply a regression-based cost-effectiveness analysis. Individual-level net benefit will be regressed on covariates, plus a binary variable reflecting AT versus ST. The model will be analyzed using generalized linear mixed models to examine cost-effectiveness over time.

### Missing Data and Dropouts

Although treating individuals lost to follow-up as presumed smoking is a widely used strategy in smoking cessation studies (ie, intention-to-treat), there are potential problems with this approach, especially when comparing interventions with differential dropout rates [132]. Therefore, we will conduct sensitivity analyses to test for treatment efficacy, assuming different missing data mechanisms. For example, we will consider a multiple imputation approach based on smoking-related participant characteristics at baseline, as well as demographics, to account for potential missing-at-random mechanisms. We will also explore pattern-mixture and selection models to account for potential missing-not-at-random mechanisms [133].

### Power Considerations

Because an intention-to-treat approach will be used for our primary analysis, and participants lost to follow-up will be classified as smokers, our power calculation assumes a sample size of 500 (250/500, 50% per group). Power is based on the primary aim of comparing self-reported abstinence rates between AT and ST at the 12-month follow-up [134]. On the basis of findings from our AAC implementation study [24,25], we expect 5% abstinence in ST. Assuming a sample size of 250 per group, a 2-group large-sample normal approximation test of proportions with a 2-sided 0.05 significance level will have 80% power to detect an increase in abstinence of 7% in AT compared with ST. Simulation studies using similar and smaller samples have demonstrated that bootstrap resampling approaches are more powerful than other approaches for estimating indirect effects and conditional indirect effects [135-137]. Therefore, we expect to have sufficient power to detect effects.

## Results

This study was funded in 2019 and approved by the institutional review board at Moffitt Cancer Center in 2019. The intervention content has been finalized, and participant recruitment and enrollment will begin in the fall of 2021.

## Discussion

### Implications of the Study

This project is designed to evaluate a fully automated smartphone intervention for people with HIV who smoke. Participants (N=500) will be randomized to receive either ST or AT. ST participants will be connected to the Florida Quitline and receive 10 weeks of NRT. ST (referred to as AAC) was developed by our team and has been successfully implemented in numerous health systems. ST will be compared with AT, a fully AT delivery approach. AT participants will receive 10 weeks of NRT plus an interactive smartphone-based intervention that comprises individually tailored audiovisual and text content. Our primary goal is to determine whether AT performs better in terms of facilitating long-term smoking abstinence (ie, 12 months postenrollment) than ST, the more resource-intensive approach. We will also explore potential mediators and moderators and conduct economic evaluations to assess the cost and cost-effectiveness of AT compared with ST. If successful, the AT intervention could be readily and cost-effectively disseminated to HIV care facilities and to a variety of outreach programs and community-based networks targeting people with HIV who smoke.

Several components of this project are novel. Despite the high smoking prevalence among people with HIV, efficacious and sustainable treatment choices are limited [19]. A consideration in the design of this study was the potential of the intervention to have a significant public health impact, while requiring relatively modest resources and removing treatment barriers. We will recruit participants using web-based advertisements, and participants will complete screening, baseline, and follow-up assessments either on the web or over the phone. Moreover, both interventions will be delivered remotely. ST will include smoking cessation treatment delivered by the Florida Quitline, and AT will consist of a fully automated smartphone intervention with proactive and user-initiated components. AT will make use of smartphone technology, including dynamically tailored audiovisual and text content, to boost treatment intensity, while enhancing accessibility, improving treatment engagement, and limiting participant burden. Finally, conducting an RCT to assess the efficacy of AT with a comprehensive economic evaluation alongside the RCT will contribute to future smoking cessation initiatives for people with HIV and the use of mobile health.

### Limitations

This study has several limitations. First, participants will consist of people with HIV who smoke recruited from the state of Florida, which might limit the generalizability of our findings. Second, participants will be recruited using only web-based advertisements; however, we believe that using web-based (vs in-person) recruitment strategies will enhance study interest and participation, particularly during the pandemic. Third, eligible participants are required to have a smartphone with a data plan and operating system compatible with the project app. Thus, individuals who do not meet this criterion will not be included. As previously mentioned, about 85% of US adults report owning a smartphone [32], so we do not expect this to significantly affect generalizability. Moreover, we will compensate participants for costs accrued due to study-related smartphone use.

### Conclusions

There is a critical need for efficacious, cost-effective, and sustainable smoking cessation treatments for people with HIV. The AT intervention is designed to help fill this need. The scientific premise for this project is built on the following: (1) cigarette smoking among people with HIV is a pressing public health problem; (2) available cessation treatments may not be meeting the needs—in terms of reach, efficacy, sustainability, affordability, and impact—of people with HIV who smoke; (3) US smartphone ownership is almost ubiquitous; (4) smartphone treatments offer tremendous reach and may be an ideal modality for people with HIV who smoke; and (5) smartphone interventions offer great dissemination and sustainability potential owing to their widespread use.

## Appendix

Multimedia Appendix 1 Peer Review Report from the National Cancer Institute Special Emphasis Panel - Improving Smoking Cessation Interventions Among People Living with HIV (R01 and R21) AIDS (National Institutes of Health, USA).

## Abbreviations

AAC: Ask Advise Connect
AT: automated treatment
ICER: incremental cost-effectiveness ratio
NRT: nicotine replacement therapy
RCT: randomized controlled trial
REDCap: Research Electronic Data Capture
ST: standard treatment
YOLS: years of life saved
