# Supplementary material for: Evaluating the Efficacy of Automated Smoking Treatment for People With HIV: Protocol for a Randomized Controlled Trial
Source: JMIR Res Protoc. 2021 Nov 17;10(11):e33183. doi: 10.2196/33183 (PMC8663670; doi:10.2196/33183)
Supplement: Multimedia Appendix 1 [file resprot_v10i11e33183_app1.pdf]

SUMMARY STATEMENT
(Privileged Communication)

PROGRAM CONTACT:
Annette Kaufman
240-276-6706
kaufmana@mail.nih.gov

Release Date: 06/21/2019
Revised Date:

Application Number: 1 R01 CA243552-01

Principal Investigator

VIDRINE, DAMON J.

Applicant Organization: UNIVERSITY OF OKLAHOMA HLTH SCIENCES CTR

Review Group: ZCA1 RTRB-U (A1)
National Cancer Institute Special Emphasis Panel
Improving Smoking Cessation Interventions Among Pople Living with HIV (R01 & R21)
AIDS

Meeting Date: 06/06/2019
Council: AUG 2019
Requested Start: 09/01/2019
RFA/PA: CA18-027
PCC: U5TC
Dual PCC: CM/ZIS
Dual IC(s): DA, MH

Project Title: Randomized Trial of Automated Video-Assisted Smoking Treatment for People Living with HIV
SRG Action: Impact Score:25
Next Steps: Visit https://grants.nih.gov/grants/next_steps.htm
Human Subjects: 30-Human subjects involved - Certified, no SRG concerns
Animal Subjects: 10-No live vertebrate animals involved for competing appl.
Gender: 1A-Both genders, scientifically acceptable
Minority: 1A-Minorities and non-minorities, scientifically acceptable
Age: 1A-Children, Adults, Older Adults, scientifically acceptable

Project Year	Direct Costs Requested	Estimated Total Cost
1	474,473	702,522
2	465,194	688,784
3	477,329	706,751
4	475,429	703,938
5	441,198	653,254
TOTAL	2,333,623	3,455,249

ADMINISTRATIVE BUDGET NOTE: The budget shown is the requested budget and has not been adjusted to reflect any recommendations made by reviewers. If an award is planned, the costs will be calculated by Institute grants management staff based on the recommendations outlined below in the COMMITTEE BUDGET RECOMMENDATIONS section.

RESUME AND SUMMARY OF DISCUSSION: In an effort to develop cost-effective and sustainable smoking cessation interventions for people living with HIV (PLWH), Dr. Vidrine proposes to evaluate a fully automated smartphone cessation intervention for HIV+ smokers. For this purpose, the investigators compare Automated Video-Assisted Smoking Treatment (AVAST) with Standard Treatment (ST) among PLWH to determine whether AVAST performs better than ST, in terms of facilitating long-term smoking abstinence. The successful completion of the proposed project could have a significant public health impact, since the AVAST has the potential to provide low-cost, sustainable, and generalizable mobile health (mHealth) interventions among PLWH, who are at increased risk for negative health consequences, including cancers. The major strength of this application lies in the strong and promising preliminary data that demonstrate the feasibility and acceptability of the proposed intervention on smoking behaviors. The approaches are rigorously designed, including the comprehensive assessment schedule, combining weekly as well as a long-term follow-up to assess sustained cessation, the proactive and brief weekly assessment and video content, the adaptive randomization procedure, a thorough assessment of cost-effective analysis, and the self-reported questionnaire about the affect, stress, and cravings. The collaboration with the HIV clinic sites located at the University of Oklahoma and Thomas Street Health Center provides additional confidence in the feasibility of the project goals. Dr. Vidrine has a long track record of studying tobacco cessation interventions for underserved and disadvantaged populations, and the team consists of experienced and accomplished investigators and clinicians. The research environment provides excellent facilities and infrastructure for carrying out the proposed experiments. Among the few minor concerns are insufficient consideration of financially disadvantaged PLWH populations for their accessibility to smartphones, and insufficient assessment of other tobacco products such as e-cigarette. Overall, the strengths clearly outweigh the weakness, and there is a likelihood of having a high impact on the identification of more effective intervention for PLWH.

DESCRIPTION (provided by applicant): Modern antiretroviral therapy has brought about significantly increased life expectancies for persons living with HIV/AIDS (PLWH). However, the effort to reduce the occurrence of cardiovascular disease and various malignancies in this population remains an important public health priority. Arguably, the most effective way to prevent these diseases in PLWH would be to reduce the prevalence of cigarette smoking. Despite the significant need, relatively few smoking cessation trials with PLWH appear in the literature. Thus, efforts to develop and evaluate sustainable, low cost evidence-based cessation interventions for smokers with HIV are needed. Given the widespread proliferation of mobile phones, the potential of using mHealth applications to improve the reach and efficacy of cessation interventions is promising, but evidence of efficacy is lacking, particularly among PLWH. With these needs in mind, the overarching purpose of the proposed project is to evaluate a fully automated smartphone intervention for HIV+ smokers. Participants (n=500) will be randomized to one of two treatment conditions: 1) Standard Treatment (ST, n=250) or Automated Video-Assisted Smoking Treatment (AVAST, n=250). ST participants will be electronically connected to state quitline- delivered cessation treatment, consisting of proactive phone counseling plus nicotine replacement therapy (NRT) in the form of transdermal patches. This approach, frequently referred to as Ask Advise Connect (AAC), was developed by our team and has been successfully implemented in numerous health systems. ST will be compared to AVAST, a fully automated treatment delivery approach. In the AVAST condition, participants will receive NRT plus an interactive smartphone-based intervention that comprises individually tailored audio/video and text content. Foundational programming and developmental work for AVAST has already been completed by the Stephenson Cancer Center's mHealth Shared Resource, and results from a series of pilot studies (including extensive work conducted at both proposed HIV clinic recruitment sites) with beta versions of the mobile application and data management system are extremely encouraging. The major goal of this project is to determine if AVAST performs better, in terms of facilitating long-term smoking abstinence (i.e., biochemically verified abstinence 12 months post-enrollment), than the more resource-intensive

ST approach. If efficacy is established, the AVAST approach will be readily adoptable by various HIV clinic and community-based organizations, and offer an efficient way to allocate limited public health resources to tobacco control interventions. The primary aim is to evaluate the efficacy of AVAST for facilitating smoking cessation among PLWH. Secondary aims include: 1) exploring potential mediators and moderators, and 2) conducting economic evaluations to assess the cost and cost-effectiveness of AVAST. In sum, there is a critical need for efficacious, cost-effective, and sustainable cessation treatments for PLWH who smoke tobacco. The proposed AVAST intervention has been designed to help fill this need.

PUBLIC HEALTH RELEVANCE: Given the extremely high rates of cigarette smoking among people living with HIV (PLWH) and the many health risks associated with smoking, developing smoking cessation treatments for PLWH is a pressing public health need. The overall purpose of this study is to evaluate a new, fully automated smartphone-delivered smoking cessation intervention for PLWH. The intervention is designed to be more effective than human- delivered counseling in helping people quit smoking, while being far less expensive.

CRITIQUE: The written critiques of individual reviewers are provided in essentially unedited form in this section. Please note that critiques and criteria scores, prepared prior to the review meeting, may not have been revised following discussions at the meeting. The "Resume and Summary of Discussion" section summarizes the final opinions of the review committee.

CRITIQUE 1

Significance:	1
Investigator(s):	1
Innovation:	3
Approach:	3
Environment:	1

Overall Impact: This application proposes an N = 500 randomized trial comparing Standard Treatment (ST; NRT, SmokefreeTXT, and proactive quitline referral) to Automated Video-Assisted Smoking Treatment (AVAST; NRT, tailored texting via a smartphone app, and weekly tailored videos) for PLWH. Strengths are numerous, and include an exceptionally strong team with prior experience in smoking cessation with PLWH, as well as in other areas critical to the proposed study; access to two large infectious diseases clinics, at which the proposed team has a prior recruitment history; a strong theoretical basis; testing of a proactive, tailored, and highly pragmatic and scalable intervention approach against a credible control condition; and strong pilot data. Weaknesses are relatively minor and do not substantially decrease enthusiasm.

1. Significance
Strengths
- This application directly addresses the important challenge of smoking cessation among PLWH, and does so in a creative and scalable way.
- The proposed trial is well supported by preliminary data while also being a clearly needed next step for the proposed intervention/technology.

Weaknesses
- None noted.

2. Investigator(s)
Strengths
- PI Dr. Damon Vidrine is a recognized tobacco cessation researcher with a strong record of productivity in that area, including significant work with PLWH.

- Dr. Businelle is one of the key developers of the platform on which the proposed app is built. The team also includes a statistician, health economist, three physicians, and two additional investigators with expertise related to the control intervention.
- Dr. Drevets is Chief of the Infectious Diseases Section at one of the proposed recruitment sites.

Weaknesses

- The specific roles/duties of the large group of co-investigators are not clear in all cases. Some overlap appears to be present.

3. Innovation

Strengths

- The proposed mobile phone app uses a unique process of weekly tailoring to produce video content that is appropriate to that participant's current status. The app itself is also used to provide tailored text messages, a process that can allow greater security than typical SMS messages.
- The proactive, brief nature of the weekly assessment and video content is an important feature given typical rates of patient-initiated use of similar technology.
- The use of tailored messages to refer back to/promote the use of the AVAST video content is also innovative.
- The weekly assessments with subsequent in-person follow-up will produce a great deal of useful data on within-individual and between-group change.

Weaknesses

- The proposed intervention content itself appears to be of high quality but not highly innovative.

4. Approach

Strengths

- The proposed intervention content and delivery approach are based have a strong theoretical basis, with hypothesized mechanisms that will be carefully measured as part of this trial.
- The control condition selected for this trial is highly credible, essentially being made up of the Ask, Advise, and Connect (to Quitline) model developed by this research team, plus NRT as well as SmokefreeTXT (to more accurately balance the text messages provided in the AVAST condition).
- The AVAST intervention will include attention to stress and negative affect, each of which are important in smoking cessation, particularly with PLWH.
- Pilot data are available suggesting good feasibility and acceptability of the AVAST app, as well as some early signal of efficacy.
- The AVAST app was originally developed—and will be further modified in the present study—using a user-centered design process.
- The assessment schedule is strong, combining weekly (during treatment) as well as long-term follow-up (notably, designed to coincide with clinic visits).
- The analytic plan includes an adjustment for multiple comparisons, and the intent-to-treat analysis focuses on sensitivity analysis and imputation rather than the suboptimal assumption that any lost to follow-up are actively smoking. (Unfortunately, however, this strength is unclear, as the application later indicates that the investigators will, in fact, consider those lost to follow-up as positive.)

Weaknesses

- E-cigarettes are not sufficiently mentioned or measured, and breath CO will not adequately detect e-cigarette use.
- There does not appear to be a plan to seek access to Quitline data for participants, which could potentially provide important additional data.
- There does not appear to be an adequate plan to obtain data regarding HIV- or other health-related data from patient medical records.

5. Environment
Strengths
- The investigators have access to two large infectious diseases clinics, at which the proposed team has a prior recruitment history.
- The University of Texas Health Sciences Center and the University of Oklahoma Health Sciences Center have adequate resources to support the proposed research.

Weaknesses
- None noted.

Study Timeline:
Strengths
- The proposed timeline is feasible and includes attention to key aspects of the proposed trial.

Weaknesses
- None noted.

Protections for Human Subjects: Acceptable Risks and/or Adequate Protections
- Adequate protections are in place for this generally low-risk study. Comment 1: additional discussion is needed regarding text messages and their security, particularly if any mention of HIV status or related issues will be included (there are no assurances that this information will not be included). Comment 2: the application indicates that it does not fall under the Single IRB rule and does not include a Single IRB plan. As a two-site study, it does appear to fall under that requirement.

Data and Safety Monitoring Plan (Applicable for Clinical Trials Only): Acceptable
- The PI will be responsible for monitoring and reporting of SAEs.

Inclusion of Women, Minorities and Children:
- Sex/Gender: Distribution justified scientifically
- Race/Ethnicity: Distribution justified scientifically
- For NIH-Defined Phase III trials, Plans for valid design and analysis: Not applicable
- Inclusion/Exclusion of Children under 18: Excluding ages <18; justified scientifically
- Children under the age of 18 will not be included due to the use of NRT and the need for alternate intervention approaches with adolescents. The investigators estimate that approximately 30% of participants will be women and 606 will be racial/ethnic minorities.

Resource Sharing Plans: Acceptable
- De-identified data will be shared in accordance with NIH requirements.

Budget and Period of Support: Recommend as Requested
- The need for three full-time coordinators/RAs and 8 Co-Investigators is not sufficiently justified.

CRITIQUE 2

Significance:	3
Investigator(s):	1
Innovation:	3
Approach:	3
Environment:	1

Overall Impact: The purpose of this project is to test an automated video-assisted smoking treatment (AVAST) compared to standard treatment (ST) among people living with HIV (PLWH) who are current smokers and interested in quitting. Smoking rates continue to be high in PLWH and, in turn, this population is at risk for significant negative health consequences. Low-cost, sustainable approaches to helping PLWH quit smoking could have a significant public health impact. This project is innovative in that it would be one of the first mHealth approaches to addressing smoking cessation in PLWH. The investigative team is excellent and demonstrates the necessary expertise to successfully complete this project. Specifically, the pilot work involving the developing and feasibility testing of the AVAST intervention was compelling, including its very promising smoking outcomes. The proposed trial is rigorously designed and includes many strengths including 2 different treatment sites, battery of assessments, long-term follow-ups, cost-effectiveness analysis, and tailored intervention content based on weekly assessments of relevant variables. Relative minor weaknesses include the need for a clearer understanding of how behavioral theories informed intervention development and intervention content is specifically hypothesized to change proposed mediators, and lack of assessment of other tobacco products. Overall, the strengths of this excellent application outweigh these relative weaknesses.

1. Significance

Strengths

- Persons living with HIV (PLWH) who smoke cigarettes are at increased risk of negative health-related consequences.
- While general population rates of cigarette smoking have decreased, high rates of cigarette smoking continue in PLWH.
- mHealth approaches for addressing smoking cessation may be cost-effective and reach a larger number of PLWH.
- Rather than tailoring specific subsets of PLWH, the proposed AVAST intervention is designed to be relevant to the broader group of PLWH who smoke.

Weaknesses

- While the tailoring of video messages based on weekly assessments is a strength of the AVAST intervention, it is not clear how the algorithm would work. More specific information on how the AVAST intervention would influence the proposed mediators would have strengthened the significance.
- Hypotheses related to the effect of the AVAST intervention on HIV-related outcomes were not adequately included.

2. Investigator(s)

Strengths

- The principal investigator, Dr. Vidrine, is the Director of Intervention Research at the Oklahoma Tobacco Research Center at the Stephenson Cancer Center. Dr. Vidrine has a long history of NIH-funded research in the area of tobacco use in special populations including PLWH.
- The rest of investigative team is impressive and has expertise in relevant areas including: intervention development and implementation (J. Vidrine, Gillapsy), HIV (Shahani, Drevets, Bui), mHealth (Businelle), and biostatistics (Frank-Pearce, Shih).

Weaknesses

- None.

3. Innovation

Strengths

- This would be one of the first self-contained, fully automated mHealth approach to addressing smoking cessation among PLWH.

Weaknesses

- A number of mHealth approaches for smoking cessation approaches already exists and it is not clear how AVAST would significantly differ from these, as it does not include any HIV-specific content.

4. Approach
Strengths

- The pilot data was impressive. The investigator conducted an open pilot followed by a small RCT of the AVAST intervention. They demonstrated the feasibility and preliminary efficacy.
- The proposed trial will be conducted across 2 different treatment sites.
- The intervention periods extend to 6 months (greater than the more typical 12 weeks).
- Plans to promote NRT adherence as part of the AVAST intervention.
- The proposed assessment battery seems appropriate (though unrealistic to expect that it can be completed in 15 minutes).
- Intervention content in the AVAST intervention will be informed by the self-reported weekly responses to questions about affect, stress, and cravings.
- The proposed sample size is based on reasonable estimates of abstinence at 12-months.
- The inclusion of cost-effective analyses is a strength.
- Sex as a biological variable will be examined in relation to outcomes.
- Relevant variables will be considered during randomization to ensure equal representation across conditions.

Weaknesses

- The conceptual framework for the proposed intervention development was non-specific. It would have been helpful to understand which theories the investigative team utilized to help inform their conceptual model.
- If the proposed mediators for the 2 interventions are the same and it is expected that the effects of the intervention on the mediators (and on outcomes) will be larger for the AVAST condition. However, it is not clear WHY the investigators expect AVAST to do better (i.e., have larger effects) if the proposed mechanisms are the same between the 2 conditions. For example, what it is about AVAST that would decrease stress more than ST?
- It does not appear that other tobacco products will be examined.

5. Environment

Strengths

- The environment is excellent and sufficiently resourced to allow for the successful completion of the proposed project.

Weaknesses

- None.

Study Timeline:
Strengths

- The Timeline appears to be appropriate.

Weaknesses

- None.

Protections for Human Subjects: Acceptable Risks and/or Adequate Protections

Data and Safety Monitoring Plan (Applicable for Clinical Trials Only): Acceptable

Inclusion of Women, Minorities and Children:

- Sex/Gender: Distribution justified scientifically
- Race/Ethnicity: Distribution justified scientifically
- For NIH-Defined Phase III trials, Plans for valid design and analysis: Not applicable
- Inclusion/Exclusion of Children under 18: Excluding ages <18; justified scientifically
- Acceptable

Resource Sharing Plans: Acceptable

Budget and Period of Support: Recommend as Requested

CRITIQUE 3

Significance:	2
Investigator(s):	2
Innovation:	2
Approach:	2
Environment:	2

Overall Impact: This application proposes to further develop and test the impact of an innovative fully automatic smart phone-based smoking cessation intervention featuring tailored video messages, among other elements. The intervention methods and, to some extent, specific content could be widely generalizable at low cost to other locations. The well-balanced research team is experienced in smoking cessation interventions designed for disadvantaged populations, including PLWH. They have conducted a series of relevant studies, including pilot studies of the proposed intervention that yielded promising results. These pilots demonstrated the feasibility, acceptability, and potential impact of the proposed intervention on smoking behaviors. The conceptual approach is well-defined and incorporated into plans for intervention content and assessments. This framework provides testable hypotheses concerning the possible mechanisms of intervention effects. Strengths of the main trial design include a relatively strong standard treatment control and a long-term 12 month follow-up period for sustained cessation and other primary outcomes. Statistical power, data analysis, and other elements of the trial design appear to be appropriate. An economic analysis is well-developed and adequately supported. Limitations of the application plans included a decision to distribute cell phones to potential participants lacking an appropriate phone. Information about the development and specific content of the video library was limited. These limitations do not substantially diminish enthusiasm for the overall approach. On balance, this is a promising intervention that is at a relatively high level of development and well-suited for testing in a larger-scale clinical trial with potential for high impact.

1. Significance

Strengths

- Cigarette smoking cessation is a high priority for improving life expectancy among PLWH.
- Evidence is lacking for effective, sustainable and generalizable interventions to address this issue.

- Smart phone-based interventions, such as text systems, are promising. There is an opportunity to advance the impact of low-cost, generalizable phone-based cessation systems through capabilities such as tailored video messages.
- The reach and population impact would be high for a successful intervention that connected smokers with an attractive and effective automated phone-based system.

Weaknesses

- Although smart phone ownership is widespread, it is less accessible to disadvantaged populations among whom smoking rates are higher. The projected smartphone ubiquity may not be realized. and some PLWH who smoke may be logistically excluded from cessation services if an automated phone service is the only option.
- Creation of a single set of video messages that are attractive to many audiences may be more challenging than creation of widely attractive text messages. Visual and audio style elements may have strong effects on message recipient responses across target population subgroups.

2. Investigator(s)
Strengths

- The Principal Investigator is experienced in design and evaluation of tobacco cessation interventions for underserved and disadvantaged populations, including PLWH, and with mobile phone tobacco cessation interventions and assessments. He has substantial experience in leading externally funded projects in these areas. He is very well qualified for his role.
- The Co-Investigators have strong qualifications and are appropriately funded for their roles in the project focused on intervention design and implementation, participant recruitment and retention, clinical issues, and evaluation.
- The Co-I located at the secondary recruitment site and the investigator focused on economic evaluation of the intervention are well-qualified.
- This is a balanced and experienced team, many of whom have collaborated on previous related work.

Weaknesses

- None noted.

3. Innovation
Strengths

- The tailored video components of the phone-based intervention will be innovative.
- The proactive and user imitated features also may be innovative.

Weaknesses

- This is an incremental advance based on substantial prior research and experience for most elements of the proposed intervention.

4. Approach
Strengths

- Study sites likely have sufficient numbers of potential participants.
- The research group has established a working relationship with the proposed sites.
- The team has conducted a substantial amount of preliminary research directly related to the proposed study.
- Preliminary studies comparing various cessation modalities to a pilot version of the proposed intervention suggest that this approach is technically feasible, acceptable and possibly more efficacious than a standard treatment.

- The conceptual approach is well-developed and details hypothesized testable mechanisms of action for the interventions.
- Recruitment, enrollment, randomization, and participant retention procedures follow established protocols that have been implemented previously at these sites or are documented in the clinical trials literature.
- An adaptive randomization procedure will contribute to balance between study groups.
- The design will compare the new treatment to a standard treatment to assess the possible advantage of the new treatment over a standard.
- The standard treatment will be delivered by the same contractor at both sites, ensuring reasonably identical treatment for participants from both sites in this study arm.
- The standard treatment will be relatively powerful.
- The underlying technology infrastructure for the tailored content interventions appears to be well-developed.
- The intervention will undergo an additional intensive pilot/feedback procedure to refine components and processes prior to the main study.
- The major outcome is verified abstinence at 12 months, a relatively strong test of sustained intervention impact.
- Statistical design and statistical power considerations are thoroughly described and appropriate. The sample size is adequate to detect a clinically relevant effect size for the major outcomes.
- The economic analysis of results will contribute to understanding of dissemination potential.

Weaknesses

- Description of the video library messages is limited, including methods used to develop or acquire them, numbers of messages available, and prior audience testing of these messages.
- Distribution of cell phones to potential participants without an appropriate phone for the interventions is unrealistic if such an automated intervention is disseminated. This feature is a limitation of generalizability.
- Respondent burden for the assessments may be high and could negatively affect participant retention.

5. Environment

Strengths

- The primary research home at Oklahoma University Health Sciences Center has a strong set of resources relevant to the proposed project and is a supportive environment for this type of research. Existing facilities and personnel for mHealth applications are a particularly important resource.
- The secondary clinical site in Houston has resources appropriate to its role in the project.
- Economic analyses will be done at M.D. Anderson Cancer Center, a research-supportive organization.

Weaknesses

- None noted.

Study Timeline:
Strengths

- Adequate time allowed for the major study phases.

Weaknesses

- None noted.

Protections for Human Subjects: Acceptable Risks and/or Adequate Protections

- Protections, risks, and benefits are assessed in detail and are adequate. Potential benefits far outweigh potential risks.

Data and Safety Monitoring Plan (Applicable for Clinical Trials Only): Acceptable

- Well-developed plan.

Inclusion of Women, Minorities and Children:

- Sex/Gender: Distribution justified scientifically
- Race/Ethnicity: Distribution justified scientifically
- For NIH-Defined Phase III trials, Plans for valid design and analysis:
- Inclusion/Exclusion of Children under 18: Excluding ages <18; justified scientifically
- Issues are discussed in detail and decisions are well justified.

Resource Sharing Plans: Acceptable

Budget and Period of Support: Recommend as Requested

THE FOLLOWING SECTIONS WERE PREPARED BY THE SCIENTIFIC REVIEW OFFICER TO SUMMARIZE THE OUTCOME OF DISCUSSIONS OF THE REVIEW COMMITTEE, OR REVIEWERS' WRITTEN CRITIQUES, ON THE FOLLOWING ISSUES:

PROTECTION OF HUMAN SUBJECTS: ACCEPTABLE

INCLUSION OF WOMEN PLAN: ACCEPTABLE

INCLUSION OF MINORITIES PLAN: ACCEPTABLE

INCLUSION OF CHILDREN PLAN: ACCEPTABLE

COMMITTEE BUDGET RECOMMENDATIONS: The budget was recommended as requested.

Footnotes for 1 R01 CA243552-01; PI Name: Vidrine, Damon J.

NIH has modified its policy regarding the receipt of resubmissions (amended applications). See Guide Notice NOT-OD-14-074 at http://grants.nih.gov/grants/guide/notice-files/NOT-OD-14-074.html. The impact/priority score is calculated after discussion of an application by averaging the overall scores (1-9) given by all voting reviewers on the committee and multiplying by 10. The criterion scores are submitted prior to the meeting by the individual reviewers assigned to an application, and are not discussed specifically at the review meeting or calculated into the overall impact score. Some applications also receive a percentile ranking. For details on the review process, see http://grants.nih.gov/grants/peer_review_process.htm#scoring.

MEETING ROSTER

National Cancer Institute Special Emphasis Panel
NATIONAL CANCER INSTITUTE
Improving Smoking Cessation Interventions Among Pople Living with HIV (R01 & R21)

ZCA1 RTRB-U (A1)
06/06/2019

Notice of NIH Policy to All Applicants: Meeting rosters are provided for information purposes only. Applicant investigators and institutional officials must not communicate directly with study section members about an application before or after the review. Failure to observe this policy will create a serious breach of integrity in the peer review process, and may lead to actions outlined in NOT-OD-14-073 at https://grants.nih.gov/grants/guide/notice-files/NOT-OD-14-073.html and NOT-OD-15-106 at https://grants.nih.gov/grants/guide/notice-files/NOT-OD-15-106.html, including removal of the application from immediate review.

CHAIRPERSON(S)

MERMELSTEIN, ROBIN J., PHD
DISTINGUISHED PROFESSOR
DEPARTMENT OF PSYCHOLOGY
UNIVERSITY OF ILLINOIS AT CHICAGO
CHICAGO, IL 60608

MEMBERS

ABRANTES, ANA M, PHD
PROFESSOR
DEPARTMENT OF PSYCHIATRY AND HUMAN BEHAVIOR
ALPERT MEDICAL SCHOOL, BUTLER HOSPITAL
BROWN UNIVERSITY
PROVIDENCE, RI 02906

BERGEN, ANDREW WELLS, PHD
SENIOR SCIENTIST
OREGEON RESEARCH INSTITUTE
EUGENE, OR 97403

CUPERTINO, PAULA, PHD
SENIOR SCIENTIST
DEPARTMENT OF BIOMEDICAL RESEARCH
HACKENSACK MERIDIAN SCHOOL OF MEDICINE
AT SETON HALL UNIVERSITY
EDISON, NJ 08837

ERBLICH, JOEL, PHD
PROFESSOR
DEPARTMENT OF PSYCHOLOGY
CITY UNIVERSITY OF NEW YORK
NEW YORK , NY 10065

FERNANDER, ANITA F., PHD
ASSOCIATE PROFESSOR
DEPARTMENT OF BEHAVIORAL SCIENCE
UNIVERSITY OF KENTUCKY COLLEGE OF MEDICINE
LEXINGTON, KY 40536

FLYNN, BRIAN S., SCD
EMERITUS PROFESSOR
DEPARTMENT OF PSYCHIATRY
OFFICE OF HEALTH PROMOTION RESEARCH
UNIVERSITY OF VERMONT
BURLINGTON, VT 05405

IBRAHIM, JENNIFER K, PHD
ASSOCIATE DEAN FOR ACADEMIC AFFAIRS
DEPARTMENT OF PUBLIC HEALTH
TEMPLE UNIVERSITY
PHILADELPHIA, PA 19122

JOSEPH, ANNE, MD
PROFESSOR OF MEDICINE
SCHOOL OF MEDICINE
UNIVERSITY OF MINNESOTA
MINNEAPOLIS, MN 55455

KATZ, DAVID A, MD
ASSOCIATE PROFESSOR
DEPARTMENT OF INTERNAL MEDICINE
UNIVERSITY OF IOWA
IOWA CITY, IA 52242

MAZIAK, WASIM, PHD, MD
CHAIR AND PROFESSOR
DEPARTMENT OF EPIDEMIOLOGY
FLORIDA INTERNATIONAL UNIVERSITY
MIAMI, FL 33199

MCCARTHY, DANIELLE ERIN, PHD
ASSOCIATE PROFESSOR
CENTER FOR TOBACCO RESEARCH AND INTERVENTION
DEPARTMENT OF MEDICINE
UNIVERSITY OF WISCONSIN SCHOOL OF MEDICINE
MADISON, WI 53711

MUILENBURG, JESSICA L., PHD
ASSOCIATE PROFESSOR AND GRADUATE COORDINATOR
DEPARTMENT OF HEALTH PROMOTION AND BEHAVIOR
COLLEGE OF PUBLIC HEALTH
UNIVERSITY OF GEORGIA
ATHENS, GA 30602

MURPHY, SHARON E, PHD
PROFESSOR
DEPARTMENT OF BIOCHEMISTRY,
MOLECULAR BIOLOGY AND BIOPHYSICS
COLLEGE OF BIOLOGICAL SCIENCES
UNIVERSITY OF MINNESOTA CANCER CENTER
MINNEAPOLIS, MN 55455

O'CLEIRIGH, CONALL MICHAEL, PHD
ASSOCIATE PROFESSOR, DIRECTOR, BEHAVIORAL MEDICINE, DEPARTMENT OF PSYCHIATRY, ASSOCIATE DIRECTOR, BEHAVIORAL SOCIAL SCIENCE CORE
HARVARD UNIVERSITY CENTER FOR AIDS RESEARCH
MASSACHUSETTS GENERAL HOSPITAL
HARVARD MEDICAL SCHOOL
BOSTON, MA 02114

ONDERSMA, STEVEN J, PHD
PROFESSOR
DEPARTMENT OF PSYCHIATRY & BEHAVIORAL NEUROSCIENCE
AND OBSTETRICS & GYNECOLOGY
MERRILL PALMER SKILLMAN INSTITUTE
WAYNE STATE UNIVERSITY SCHOOL OF MEDICINE
DETROIT, MI 48236

OSTROFF, JAMIE S, PHD
PROFESSOR, VICE CHAIR FOR RESEARCH
CHIEF, BEHAVIORAL SCIENCES SERVICE
DEPARTMENT OF PSYCHIATRY
AND BEHAVIORAL SCIENCES
MEMORIAL SLOAN-KETTERING CANCER CENTER
NEW YORK, NY 10065

PATTEN, CHRISTI A, PHD
PROFESSOR
DEPARTMENT OF PSYCHIATRY
AND PSYCHOLOGY
MAYO CLINIC
ROCHESTER, MN 55905

STANTON, CASSANDRA A, PHD
ADJUNCT PROFESSOR
DEPARTMENT OF ONCOLOGY
LOMBARDI COMPREHENSIVE CANCER CENTER
GEORGETOWN UNIVERSITY MEDICAL CENTER
WASHINGTON, DC 20007

SUTFIN, ERIN L, PHD
ASSOCIATE PROFESSOR AND VICE CHAIR
DIVISION OF PUBLIC HEALTH SCIENCES
DEPARTMENT OF SOCIAL SCIENCES AND HEALTH POLICY
SCHOOL OF MEDICINE
WAKE FOREST UNIVERSITY
WINSTON-SALEM, NC 27157

WILSON, STEPHEN JEFFREY, PHD
ASSOCIATE PROFESSOR
DEPARTMENT OF PSYCHOLOGY
PENNSYLVANIA STATE UNIVERSITY
UNIVERSITY PARK, PA 16801

SCIENTIFIC REVIEW OFFICER

LEE, BYEONG-CHEL, PHD
SCIENTIFIC REVIEW OFFICER
RESOURCES AND TRAINING REVIEW BRANCH
DIVISION OF EXTRAMURAL ACTIVITIES
NATIONAL CANCER INSTITUTE
NATIONAL INSTITUTES OF HEALTH
ROCKVILLE, MD 20850

EXTRAMURAL SUPPORT ASSISTANT

TIONG, KATHY
PROGRAM ANALYST
OFFICE OF THE DIRECTOR, DEA
NATIONAL CANCER INSTITUTE
NATIONAL INSTITUTES OF HEALTH
BETHESDA, MD 20892

PROGRAM REPRESENTATIVE

KAUFMAN, ANNETTE R, PHD, MPH
HEALTH SCIENTIST/PROGRAM DIRECTOR
TOBACCO CONTROL RESEARCH BRANCH
NATIONAL CANCER INSTITUTE
NATIONAL INSTITUTE OF HEALTH
ROCKVILLE, MD 20892

Consultants are required to absent themselves from the room during the review of any application if their presence would constitute or appear to constitute a conflict of interest.
